# Sedentary lifestyle and its associated factors among adolescents from public and private schools of a Brazilian state capital

**DOI:** 10.1186/s12889-016-3836-9

**Published:** 2016-11-21

**Authors:** Flávia Miquetichuc Nogueira Nascente, Thiago Veiga Jardim, Maria do Rosário Gondim Peixoto, Carolina de Souza Carneiro, Karla Lorena Mendonça, Thaís Inácio Rolim Póvoa, Ana Luiza Lima Sousa, Weimar Kunz Sebba Barroso, Paulo César Brandão Veiga Jardim

**Affiliations:** Hypertension League, Federal University of Goias, Goiania, Brazil

**Keywords:** Adolescents, Physical Activity, Sedentary lifestyle

## Abstract

**Background:**

Adolescence is a transition stage between childhood and adulthood and is an important phase for the acquisition of future lifestyles, including the practice of physical activity (PA). The prevalence of sedentary lifestyle in adolescents is often high, creating the need for studies addressing the practice of PA and its associated factors for a better understanding of the phenomenon and possible interventions that would encourage positive changes.

**Methods:**

Cross-sectional study of a representative sample of students aged 14–18 years enrolled in both public and private schools of a large Brazilian city to determine the level of physical activity (PA) and its associated factors. Sedentary lifestyle was measured by applying the International Physical Activity Questionnaire. The independent variables were gender, age, race, tobacco use and alcohol consumption in the past 30 days, socioeconomic status, body mass index, waist circumference and blood pressure. The crude prevalence ratio was used as a measure of association and was estimated from a Poisson regression.

**Results:**

The sample consisted of 862 adolescents with a mean age of 15.4 ± 1.1 years. Females were predominant (52.8%), and the age between 14 and 15 years was the most frequent (52.2%). The majority of the group reported themselves as Caucasians (51.2%), belonging to socioeconomic class C (52.5%) and were attending to public schools (69.1%). The prevalence of sedentary lifestyle was 66.8% (95% confidence interval [CI]: 63.5–69.9), where values of 65.4% and 69.9% were observed among students from public and private schools, respectively (*p* = 0.196). Sedentary lifestyle was more frequent in females (78.0% vs 54.3%; *p* < 0.001). The factor directly associated with sedentary lifestyle was female gender both in public and private schools and the only independent variable related to sedentarism was also female gender.

**Conclusion:**

The prevalence of sedentary lifestyle was extremely high in the population of adolescents studied both in public and private schools. Female sex was directly associated with sedentary lifestyle.

## Background

Adolescence is a transition stage between childhood and adulthood and is an important phase for the acquisition of future lifestyles, including the practice of physical activity (PA) [1]. In this period, the PA level decreases with increasing age and is a strong predictor of this practice in adulthood [2].

The benefits obtained with the regular practice of PA involve cardiovascular, metabolic and musculoskeletal functions and also represent an important role for reducing body fat [3]. As a result, PA reduces the risks of developing diseases associated with sedentary lifestyle, such as coronary heart disease, hypertension, obesity, type 2 diabetes, osteoporosis, colon cancer, depression, impaired glucose tolerance and lipid abnormalities [4].

Some national [5] and international [6] studies report the importance of the regular practice of PA as an essential habit to prevent chronic diseases. The studies state that this practice should be encouraged during adolescence so that it will have a greater chance of being maintained during adulthood.

According to PA guidelines, moderate to vigorous PA lasting at least 30 min a day and at least 5 days a week is required to obtain health benefits [7, 8].

The results obtained with Brazilian adolescents are discrepant and indicate that the prevalence of sedentary lifestyle ranges from 3.0 to 64.3%, depending on the cut-off point and the instruments used for measuring this parameter [9, 10].

Despite the differences due to different methodologies used to assess this practice, the prevalence of sedentary lifestyle in adolescents is often high, creating the need for studies addressing the practice of PA and its associated factors for a better understanding of the phenomenon and possible interventions that encourage positive changes.

Accordingly, this study aimed to estimate the prevalence of sedentary lifestyle and its associated factors among adolescents enrolled in public and private schools of a large city of the Midwest region of Brazil. The study hypothesis is that potential modifiable factors related to sedentarism can be identified in this population therefore allowing health policy changes as well as interventions aiming to reduce this increasingly common situation among adolescents.

## Methods

This school-based cross-sectional study was part of a main epidemiological project named: “Home blood pressure measurement and its correlation with left ventricle mass index and insulin resistance in adolescents with white coat and masked hypertension” performed with secondary school students from both public and private schools of the city of Goiânia, state of Goiás, Brazil, in 2011. Goiânia has a population of 1,302,001 inhabitants and is located in the Midwest region of Brazil; its land area is 732.80 km^2^, and its population density is 1776.75 hab/km^2^ [11].

This study was approved by the Research Ethics Committee of the Institution. The eligible adolescents who agreed to participate in the study signed an informed consent form and so did their guardians; the adolescents were then asked to complete the questionnaires and were submitted to anthropometric and blood pressure (BP) assessments.

The initial sample size calculation considered a population of 133,528 students enrolled in both public and private schools of Goiânia [11], the prevalence of masked hypertension (7%) and white-coat hypertension (10%) [12], an absolute error of 2% and a confidence level of 95%. These parameters determined that a sample size of 1024 students would be required. A total of 1169 students were analysed (10% more than the number required), 862 of whom were aged 14–18 years (inclusion criterion).

Considering a prevalence of sedentary lifestyle of 62.5% among the adolescents [9] and using a 95% confidence interval (CI), this sample size allows the estimation of the prevalence of sedentary lifestyle with a margin of error of 3.5%, in addition to detecting a correlation of approximately 0.20, considering α = 0.05 and β = 0.10 [13].

All public and private schools from the 9 regions of the city were identified. After that 5 public and 5 private schools from each region were drawn and then invited to participate. The school contact followed the drawing order. At the end 26 schools agree to participate (17 public and 9 private). The adolescents selection was performed by random drawing of the students enrolled in the institutions, stratified by age and gender.

The exclusion criteria were adolescents with physical disabilities that prevented the practice of PA; those aged < 14 and > 19 years; pregnant women; those with disabling chronic diseases and those who were on continuous medication.

Data was collected by a trained team over 13 months. The team consisted of four supervisors and nine data collectors (7 interviewers and 2 anthropometrists) who were graduate students of the Physical Therapy and Nutrition programs and had been previously trained.

This study used a standardised questionnaire that included the International Physical Activity Questionnaire (IPAQ), anthropometric measurements (height, weight and waist circumference) and BP measurements according to the methodology recommended by the 4^th^ Task Force [14]. This questionnaire provided information about school type (public or private); adolescent identification (name, date of birth, age, gender, skin colour, telephone number and address); personal history (birth and height at birth, prematurity, menarche, current diseases, medications); family history (hypertension, diabetes, obesity, premature cardiovascular disease); tobacco use and alcohol consumption; usual PA (IPAQ); diet habits; physical examination (weight, height, waist circumference [WC], BP and heart rate) and socioeconomic status (classes A, B, C, D, and E in descending order according to the Brazilian Association of Research Companies) [15].

The nutritional status of adolescents was assessed using body mass index (BMI = weight [kg]/height [m]^2^). Underweight or normal weight students were grouped as “normal weight”; those students who were overweight or obese were considered “overweight” (above the 85th percentile of BMI cut-off points, specific for age and gender) [16].

The cut-off points for WC were adjusted by age and gender according to Freedman’s classification that establish the 90th percentile, adjusted for gender, as an indicator of metabolic changes [17].

BP was considered altered in those adolescents who presented BP measurements (systolic and/or diastolic) at or above the 90th percentile for the corresponding age, gender and height [14].

The outcome variable was “sedentary lifestyle”, defined as a moderate and/or vigorous practice of PA less than 300 min per week [2, 3, 18]. Therefore light physical activity (walking) was not considered for the classification of sedentary lifestyle.

The short-form (Version 8) of the IPAQ was applied to estimate the prevalence of sedentary lifestyle, considering the PA practiced in the previous week as a reference [19]. The questions addressed the frequencies and durations of light (walking), moderate and vigorous PA [20, 21].

Data were entered in duplicate in Epiinfo 3.5.1 software. The data were then validated (i.e., the final database) using the same software to ensure greater reliability of the information.

The variables (outcome and covariates) were categorised and analysed using Stata 12.0 software. Descriptive analysis and the analysis of the association between independent variables with sedentary lifestyle were performed. Pearson’s Chi-square Test was used to evaluate the differences between school types. The significance level was set as 5% for two-tailed tests.

A crude Poisson regression was initially used to analyse the factors associated with sedentary lifestyle. Variables with p ≤ 0.20 were tested in the multivariate analysis using a Poisson regression with robust variance estimates [22]. The variables associated with the outcome (*p* < 0.05) were kept in the final model.

## Results

A total of 862 students enrolled in public schools (69.1%) and private schools (30.9%) were evaluated. The mean age was 15.4 ± 1.1 years, with a minimum of 14 and maximum of 18 years. The age group of 14–15 years was the most prevalent (51.3% in public schools and 54.1% in private schools). There were no significant differences between genders either in the total sample or when analysing the public and private schools separately.

The number of adolescents who claimed to be “non-white” was higher in the public schools when compared to private (53.2% x 39.1 – *p* < 0.001). Regarding socioeconomic classification, 63.4% of students from public schools reported belonging to Class C, while 28.2% of students from private schools reported the same (*p* < 0.001). The majority (71.0%) of the students from private schools reported belonging to classes A and B (Table 1).

**Table 1:** Characteristics of the sample of adolescents from public and private schools (*n* = 862), Goiânia, Brazil, 2010–2011

Variable	Total sample		Public School		Private School		*p* ^*a*^
	n	% (95% CI)	n	% (95% CI)	n	% (95% CI)	
School type							
Public	596	69.1 (65.9–72.2)		-		-	
Private	266	30.9 (27.8–34.1)		-		-	
Gender							
Male	407	47.2 (43.8–50.6)	280	47.0 (42.9–51.1)	127	52.3 (41.6–53.9)	0.835
Female	455	52.8 (49.3–56.1)	316	53.0 (48.9–57.1)	139	47.7 (46.1–58.4)	
Age (years)							
14–15	450	52.2 (48.8–55.5)	306	51.3 (47.2–55.4)	144	54.1 (47.9–60.2)	0.448
16–18	412	47.8 (44.4–51.1)	290	48.7 (44.6–52.8)	122	45.9 (39.8–52.0)	
Skin colour^b^							
Non-white	420	48.8 (45.4–52.2)	316	53.2 (48.9–57.0)	104	39.1 (33.1–45.2)	<0.001*
White	440	51.2 (47.7–54.5)	278	46.8 (42.6- 50.7)	162	60.9 (54.8–66.8)	
Socioeconomic classification							
A - B	375	43.5 (40.1–46.8)	186	31.2 (27.5–35.0)	189	71.0 (65.2–76.4)	
C	453	52.5 (49.1–55.9)	378	63.4 (59.4–67.2)	75	28.2 (22.9–34.0)	<0.001*
D – E	34	4.0 (2.74–5.46)	32	5.4 (3.7–7.5)	2	0.8 (0.1–2.7)	
Tobacco use							
No	848	98.4 (97.2–99.1)	584	98.0 (96.5–98.9)	264	99.3 (97.3–99.9)	0.176
Yes	14	1.6 (0.89–2.71)	12	2.0 (1.0–3.5)	2	0.7 (0.1–2.7)	
Alcohol consumption							
No	240	27.8 (24.8–30.9)	179	30.0 (26.3–33.9)	61	22.9 (18.0–28.5)	0.032*
Yes	622	72.2 (69.0–75.1)	417	70.0 (66.1–73.6)	205	77.1 (71.5–82.0)	
Sedentary lifestyle							
No	286	33.2 (30.0 - 36.4)	206	34.6 (30.7 - 38.5)	80	30.1 (24.6 - 36.0)	0.196
Yes	576	66.8 (63.5 - 69.9)	390	65.4 (61.5 - 69.2)	186	69.9 (64.0 - 75.4)	
Altered BP^c^							
No	712	82.6 (79.8–85.0)	499	83.7 (80.5–86.6)	213	80.1 (74.8–84.7)	0.192
Yes	150	17.4 (14.9–20.1)	97	16.3 (13.4–19.5)	53	19.9 (15.3–25.2)	
Altered WC^d^							
No	741	86.0 (83.4–88.2)	523	87.7 (84.8–90.2)	218	81.9 (76.8–86.3)	0.024*
Yes	121	14.0 (11.7–16.5)	73	12.3 (9.7–15.1)	48	18.1 (13.6–23.2)	
Overweight							
No	682	79.1 (76.2–81.8)	484	81.2 (77.8–84.3)	198	74.4 (69.8–79.6)	0.024*
Yes	180	20.9 (18.2–23.8)	112	18.8 (15.7–22.2)	68	25.6 (20.4–31.2)	

^a^Pearson’s chi-squared test; ^b^Two individuals did not respond; ^c^Blood pressure; ^d^Waist circumference

*Statistically significant at *p* < 0.05

Tobacco use had low prevalence rates in both school groups (2.0% in public schools and 0.7% in private schools). However, alcohol consumption was observed in approximately 70.0% of adolescents from both groups and was more prevalent among students from private schools (77.1%; *p* = 0.032) (Table 1).

**Fig. 1:**
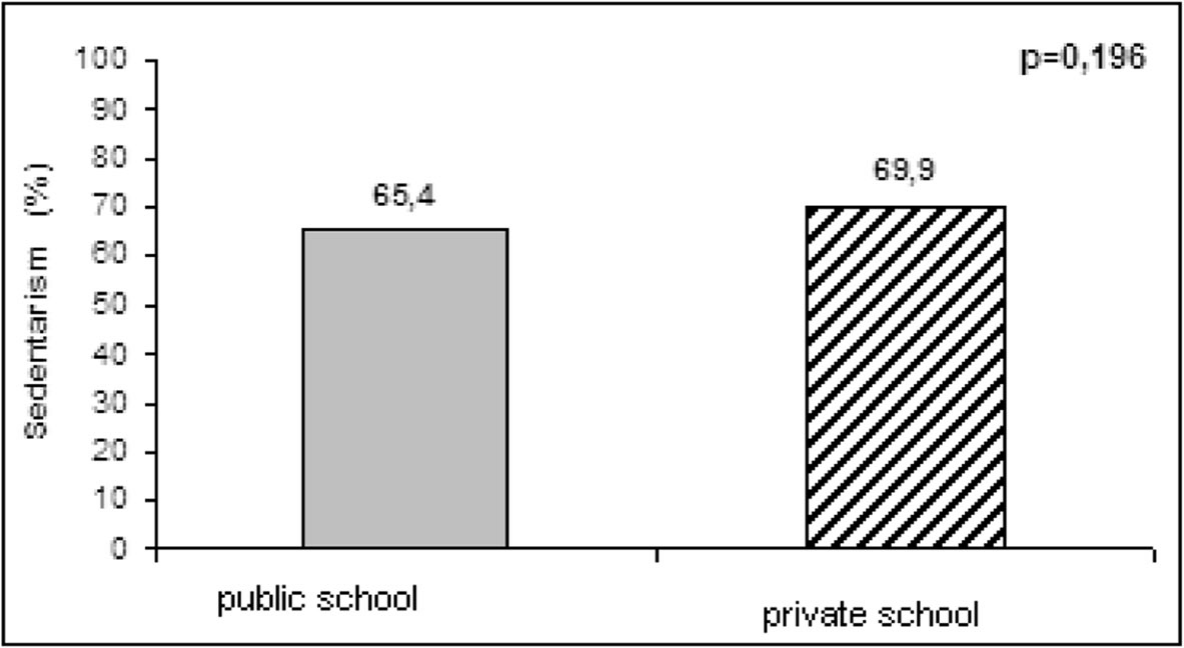
Sedentary lifestyle among adolescents from public and private schools (*n* = 862), Goiânia-GO, 2010–2011

Sedentary lifestyle was highly prevalent in the total sample (66.8%), with no significant difference (*p* = 0.196) between the students from private (69.9%) and public schools (65.4%) (Fig. 1 and Table 1).

Altered BP was observed in 17.4% of the adolescents, with no difference between school types. Altered WC was observed in 14.0% of the students, with a significant difference between school types (12.3% in public schools and 18.1% in private schools) (*p* = 0.024). In the analysis of the total sample, 20.9% of the adolescents were overweight, with a smaller number observed among the students from public schools (18.8%) compared to the private schools (25.6%; *p* = 0.024) (Table 1).

Considering the total sample, 66.8% of the adolescents were sedentary. Regarding gender, 78.0% of the females and 54.3% of the males were sedentary (*p* < 0.001).

Table 2 shows the data of the bivariate analysis with sedentary lifestyle as the outcome. Sedentary lifestyle among the students from public and private schools was positively associated with female gender. There were no significant differences for the other independent variables (Table 2).

**Table 2:** Prevalence of sedentary lifestyle among adolescents from public and private schools (*n* = 862), Goiânia, Brazil, 2010–2011

	Public School (*n* = 596)			Private School (*n* = 266)		
Variables	n (%)	Crude PR^a^	p^b^	n (%)	Crude PR^a^	P^b^
Gender						
Male	150 (53.6%)	0.70	<0.001*	71 (55.9%)	0.67	<0.001*
Female	240 (76.0%)	1		115 (82.7%)	1	
Age (years)						
14–15	204 (66.7%)	1		104 (72.2%)	1	
16–18	186 (64.1%)	0.96	0.517	82 (67.2%)	0.93	0.380
Skin colour						
Non-white	201 (63.6%)	0.94	0.303	68 (65.4%)	0.90	0.210
White	188 (67.6%)	1		118 (72.8%)	1	
Socioeconomic classification						
A - B	113 (60.8%)	1		135 (71.4%)	1	
C	254 (67.2%)	1.10	0.145	50 (66.7%)	0.93	0.462
D - E	23 (71.9%)	1.18	0.180	1 (50.0%)	0.70	0.615
Tobacco use						
No	386 (66.1%)	1		185 (70.0%)	1	
Yes	4 (33.3%)	0.50	0.095	1 (50.0%)	0.71	0.634
Alcohol consumption						
No	107 (59.8%)	1		43 (70.5%)	1	
Yes	283 (67.9%)	1.13	0.070	143 (69.8%)	0.99	0.912
Altered BP^c^						
No	332 (66.5%)	1		147 (69.0%)	1	
Yes	58 (59.8%)	0.90	0.231	39 (73.6%)	1.07	0.497
Altered WC^d^						
No	336 (64.2%)	1		155 (71.1%)	1	
Yes	54 (74.0%)	1.15	0.066	31 (64.6%)	0.91	0.405
Overweight						
No	320 (66.1%)	0.95	0.483	141 (71.2%)	0.93	
Yes	70 (62.5%)	1		45 (66.2%)	1	0.454

^a^Prevalence ratio; ^b^Poisson regression; ^c^Blood pressure; ^d^Waist circumference

*Statistically significant at *p* < 0.05

The multiple Poisson regression analysis revealed that only males exhibited a lower prevalence of sedentary lifestyle in both public and private schools (PR = 0.72; PR = 0.68; *p* < 0.001) (Table 3).

**Table 3:** Risk factor for sedentary lifestyle among adolescents from public and private schools (*n* = 862), Goiânia, Brazil, 2010–2011

	Public School			Private School		
	Adjusted PR^a^	95%CI	p^b^	Adjusted PR^a^	95%CI	p^b^
Sedentary lifestyle						
Gender						
Male	0.72	0.63–0.81	<0.001*	0.68	0.57–0.80	<0.001*
Female	1			1		

^a^Prevalence ratio; ^b^Adjusted Poisson regression

*Statistically significant at *p* < 0.05

## Discussion

This study was the first conducted in the Midwest region of Brazil with a representative sample of adolescent students from public and private schools demonstrating the prevalence of sedentary lifestyle and its association with sociodemographic variables, lifestyle, BP and anthropometric factors.

Adolescents from different schools were assessed not only to increase the representativeness of the sample but also due to potential social inequalities faced by this population and its possible influence on the investigated factors. Previously published data that investigated factors associated to physical inactivity [9] showed that as the socioeconomic level increases the prevalence of physical inactivity decreases, as well as differences in physical activity patterns related to gender, alcohol consumption and smoking.

The sedentary lifestyle classification (practice of PA for less than 300 min per week) [2, 3, 18] is universally accepted for children and adolescents. Using this methodology, this study revealed high prevalence rates of sedentary lifestyle in both the total sample (66.8%) and the assessment by school type (65.4% in public schools and 69.9% in private schools).

Studies in Brazilian cities using the same instrument (IPAQ) and the same sedentary lifestyle cut-off point corroborate the findings of this study, with high prevalence rates of sedentary lifestyle observed in the city of Maringa, state of Paraná (62.5%) [9] and São Paulo, state of São Paulo (56.9%) [23].

Other studies that used the same cut-off point also found a high prevalence of sedentary lifestyle among adolescents, for example, the National Adolescent School-based Health Survey [24], which assessed 60,973 students from Brazilian state capitals, aged 13 to 15 years. This study found a high prevalence of sedentary lifestyle (56.9%), with 57.4% in public institutions and 54.9% in private institutions. A study conducted in the capital of Santa Catarina revealed that sedentary lifestyle was observed in 62.69% of the students aged 12–18 years [25].

Similarly, other studies confirmed the findings of the present study, reporting high numbers of sedentary adolescents. A study conducted with 775 adolescents aged 14 to 19 years in a public school of a countryside city of São Paulo state revealed that 64.3% of the students were sedentary [10]. Another study with students aged 12 to 17 and enrolled in private schools of the capital of Ceará state revealed that 63.0% of them were sedentary [26].

In general, the present study demonstrated that sedentary lifestyle was more prevalent among females than among males. This difference was observed in both public and private schools (*p* < 0.001). Similar results were reported in both national [9, 27] and international studies [28, 29].

The difference observed in the practice of PA among young males and females may be explained by the fact that males practice more moderate and vigorous activities as a result of physical and cultural aspects because males are often “stronger, more virile and more courageous”. In turn, young females tend to practice lighter activities, which are justified by the fact that they are weaker, more delicate and graceful [30, 31].

A limitation of this study was the fact that it was a cross-sectional study; therefore, the association between outcome and exposure was performed only at the time of data collection.

Another potential limitation was the analysis of a population of schoolchildren, which excluded adolescents who were out of school; however, the same methodology has been applied in several European countries and other countries around the world [32, 33]. The easy access to this adolescent population and the benefits provided by the studies (such as an integrated planning between the health and education sectors) justify conducting this study. In addition, according to the Brazilian Institute of Geography and Statistics - IBGE, the education system coverage in Brazil has increased over the years, approaching universalisation [34]. Finally, the fact that the sample studied was from two strata (public and private schools) increases the representativeness of the target population.

The instrument used to measure the physical activity level (IPAQ) presents some advantages, such as being internationally and nationally validated, self-applied, low cost, widely used by a large number of individuals and having good cultural adaptability [35, 36].

The high prevalence of sedentary lifestyle among adolescents and its association with gender (in both public and private schools) are among the important findings of this study.

Although the bivariate analysis revealed no association between sedentary lifestyle and overweight and altered WC, high prevalence rates of overweight (20.0%, *p* = 0.024) and altered WC (14.0%, *p* = 0.024) were observed. Similar to another study performed with adolescents from the Brazilian city of Rio de Janeiro, higher prevalence rates of these variables were observed in private schools when compared to public schools [37].

The consumption of alcohol in the past thirty days, regardless of frequency and intensity, was used to measure alcohol consumption. Although there was no association between sedentary lifestyle and alcohol consumption, the analysis of this time frame revealed high alcohol consumption among adolescents (70.0% in public schools and 77.1% in private schools, *p* = 0.032). This finding represents an aggravating factor because it constitutes a serious public health problem, considering that the early use of alcohol is an exposure factor for health problems in adulthood, in addition to increasing the chance of these adolescents becoming potential lifelong alcohol drinkers [38].

The information obtained from the collected data represents a baseline that aims to guide integrated planning actions of the health and education sectors. It is envisaged that schools play an important role in health promotion, with intervention plans involving the reduction of tobacco use and alcohol consumption and the encouragement of the practice of PA and healthy eating habits [39].

The role of the school involves reinforcing learning and literacy, in addition to encouraging students to adopt healthy lifestyles. Therefore, when these adolescents complete their compulsory education, they may be supporters of the regular practice of PA, not only due to its preventive power but also due to the opportunity to learn how to make appropriate choices that will ensure a better quality of life over time [39, 40].

Several sectors, such as education, health, sport and social security, should adopt an integrated approach towards the encouragement of the regular practice of PA, contributing with measures that influence the knowledge about its benefits, individual preparation and availability of places with safe and pleasant access to the practice of leisure PA [40].

In addition to this general and intersectoral involvement, there is an important measure involving primary care: structured counselling for PA and the inclusion of this counselling in the therapeutic context as a key factor to prevent and treat several diseases [40].

## Conclusion

The prevalence of sedentary lifestyle was extremely high in the population of adolescents studied both in public and private schools. Female sex was directly associated with sedentary lifestyle. School based interventions should be addressed to change this scenario with a particular attention to female students.

## Abbreviations

BMI: Body mass index
BP: Blood pressure
CI: Confidence interval
IBGE: Brazilian Institute of Geography and Statistics
IPAQ: International Physical Activity Questionnaire
PA: Physical activity
WC: Waist circumference
WHO: World Health Organization
